# Description of a New GLA Gene Variant in a Patient with Hypertrophic Cardiomyopathy. Is it Fabry Disease?

**DOI:** 10.5935/abc.20190126

**Published:** 2019-07

**Authors:** Marcelo Imbroinise Bittencourt

**Affiliations:** Hospital Universitário Pedro Ernesto da Universidade do Estado do Rio de Janeiro, Rio de Janeiro, RJ - Brazil

**Keywords:** Alpha-Galactosidase/deficiency, Fabry Disease/genetics, Fabry Disease/metabolismo, cardiomyopathy, Hypertrophic, Multiple Chronic Conditions

Hypertrophic cardiomyopathy (HCM) is the most common monogenic cardiovascular disease in Brazil, with a prevalence of approximately 1:500 individuals. The autosomal dominant inheritance pattern is the most common one. More than 20 genes have been reported as being associated with the disease, and most of them encode sarcomeric proteins.^1,2^

Fabry disease (FD) is a lysosomal storage disorder caused by a-galactosidase-A deficiency due to a mutation in the *GLA* gene, with X-linked inheritance. It causes alterations in multiple organs, including the heart, mimicking HCM.^3^

In a publication by Favalli et al.,^4^ a prospective 10-year FD screening study, hypertrophy with wall thickness >13 mm was the most common finding of the disease, occurring in approximately 50% of individuals with mutations in the *GLA* gene, followed by acroparesthesia and renal failure.^4^

A better understanding of the disease prevalence has been attained as its screening has been carried out in several populations. A Chinese study published in the past decade Indicates a prevalence of 1: 1600 men.^5^ Some mutations can cause the disease with limited heart manifestations, and this has already been reported in previous studies.^6,7^ The hypertrophy pattern may be useful to differentiate FD from HCM, but it is not an easy task. In HCM, the distribution of the left ventricular hypertrophy is characteristically asymmetric and heterogeneous, with several possible phenotypes. In FD, the hypertrophy is typically concentric, without obstruction of the left ventricular outflow tract, and the tapering of the basal portion of the left ventricular posterior wall is a characteristic of the final stage of the disease.

In the current issue of the Brazilian Archives of Cardiology, Chaves-Markman et al.^8^ found mutations in the *GLA* gene in 6.7% of a cohort of 60 HCM patients and reported a new variant, c.967C>A (p.Pro323Thr), a missense mutation. This mutation has not been previously described, showing the originality of this work.^8^ Two other variants were described in this group of patients: c.937G>T (p.Asp313Tyr) and c.352C>T (p.Arg118Cys). Interestingly, three of the four patients who had mutations in the *GLA* gene were females, a phenomenon also found in the article published by Csányi B et al.,^7^ when they described the Ile239Met variant.7 This finding is little expected in patients with FD due to inactivation of the X chromosome in heterozygous women, resulting in milder symptoms.^9^

The main question of this article to be discussed is whether the new mutation is in fact responsible for the left ventricular hypertrophy. The detection of any rare missense variant represents a challenge for the conclusion of causality in any genetic condition, and FD is no different. Therefore, since only the analysis of the *GLA* gene was performed, one cannot rule out the possibility that another pathogenic variant in some other gene that causes HCM may be present in the patient in which the c.967C>A (p.Pro323Thr) variant was detected, as it was correctly highlighted in the study limitations.

Nevertheless, it is a very relevant finding, as the genetic alteration is in a highly conserved area of the protein and this has an impact on the definition of pathogenicity of a new variant. It should be emphasized that the patient had a transient ischemic attack among the extracardiac symptoms, without any documentation of atrial fibrillation, which could be associated with FD.

As emphasized by the author, the histopathological study is important for the diagnosis of FD, and it may help the groups that are discovering new variants of uncertain significance in the *GLA* gene to define its actual importance, especially in patients with the isolated cardiac form.

Every diagnostic method has its limitations, and the identification of a complex condition such as FD will increasingly require a multiple approach by integrating several modalities (genetics, biochemistry, imaging and histology), which will generate more evidence for each experiment. Definitely, the study carried out by Chaves-Markman et al. represents an important step in the genetic screening and knowledge acquisition of the association between HCM and FD in the Brazilian population.
